# Recent advances in tissue imaging for cancer research

**DOI:** 10.12688/f1000research.19037.1

**Published:** 2019-11-25

**Authors:** Oscar Maiques, Mirella Georgouli, Victoria Sanz-Moreno

**Affiliations:** 1 Barts Cancer Institute, John Vane Science Building, Charterhouse Square, Queen Mary University of London, London, EC1M 6BQ, UK; 2 Oncology Cell Therapy RU, GlaxoSmithKline, Stevenage, London, SG1 2NY, UK

**Keywords:** immunohistochemistry, digital pathology, multiplex flow cytometry, intravital imaging

## Abstract

Image analysis in clinical research has evolved at fast pace in the last decade. This review discusses basic concepts ranging from immunohistochemistry to advanced techniques such as multiplex imaging, digital pathology, flow cytometry and intravital microscopy. Tissue imaging *ex vivo* is still one of the gold-standards in the field due to feasibility. We describe here different protocols and applications of digital analysis providing basic and clinical researchers with an overview on how to analyse tissue images. *In vivo* imaging is not easily accessible to researchers; however, it provides invaluable dynamic information. Overall, we discuss a plethora of techniques that - when combined - constitute a powerful platform for basic and translational cancer research.

## Introduction

Methodology for imaging human or mouse tissues has seen great progress, ranging from basic histopathological analysis under the microscope to recent automation (computer-assisted diagnosis and machine learning technologies) and image digitalisation. In addition to clinical research, histopathology combined with imaging is a valuable tool to assess *in situ* changes in tissue specimens in an array of solid tumors. Such analysis can be either a starting point for investigation followed by dissection of cellular and molecular mechanisms or the endpoint validation of *in vitro* findings.

In this review, we discuss the recent advances in high-performance tissue imaging. We classify the tissue imaging modalities into three major groups: immunohistochemistry (IHC) (with a focus on new digital imaging techniques), multiplex flow cytometry, and intravital microscopy. We summarise the strengths and possible applications of these methods for discovery science and for translational/clinical applications.

## Immunohistochemistry and digital pathology

Observation is the first and one of the most fundamental steps in scientific methodology. Studies combining tissue dissection and microscopy are crucial in diagnosis and biomedical translational research ^1, 2^. However, accurate and quantitative image analysis still remains the main a challenge ^3^. IHC-based applications have expanded significantly over the last decade ^4^.

IHC is a method for detecting antigens or haptens in cells of a tissue section by exploiting the principle of specific antigen–antibody recognition. The antibody–antigen binding can be visualised in different ways. Enzymes, such as horseradish peroxidase (HRP) or alkaline phosphatase (AP), are commonly used to catalyse a colour-producing reaction ^5^. Furthermore, one of the key advantages of IHC is that it offers combined histopathological information (i.e. tissue necrosis, tumor load, and architecture of surrounding non-tumoral components) with biomarker expression. Molecular and clinical oncologists studying the tumour microenvironment (TME) need to better understand the heterogeneous cellular components and structure of tumours to predict therapy responses.

Multiplexed IHC (mIHC) methods have been developed to detect several proteins in a single formalin-fixed paraffin-embedded (FFPE) tissue section. Currently, several protocols have been described for staining in a multiplexed fashion. The most extended methodology combines conventional IHC protocols with imaging and image analysis, referred to as SIMPLE (sequential immunoperoxidase labelling and erasing). As implied by its name, this method involves labelling a sample with peroxidase-based indirect IHC and subsequent removal of the alcohol-soluble substrate, 3-amino-9-ethylcarbazole (AEC), combined with an acid-based antibody elution step. Several markers (antibodies) can be used after repetition of these steps. Digital reconstruction of the different markers used is then performed, and subsequently the images are overlaid and pseudocolours to each marker are assigned. Hence, SIMPLE is a useful approach to eliminate the problems associated with multiprobe colour compatibility and antigens located in the same cellular compartment. On the other hand, multiplexing may be challenging owing to compromised tissue integrity after repetitive rounds of ethanol dehydration and heat-induced antigen retrieval ^6, 7^. To address this, different protocols have been developed where tyramide signal amplification (TSA) methods are used. Tyramide is an organic phenol that can be conjugated to biotin or fluorescent labels. In the presence of a catalyst like HRP, tyramide becomes activated and covalently bound to electron-rich regions. This typically occurs on tyrosine residues in proteins, present in the vicinity of protein antigens. The covalent nature of tyramide–tyrosine engagement results in heat-mediated removal (stripping) of primary and secondary antibody pairs bound to the antigen while preserving the antigen-associated fluorescence signal. This facilitates the sequential use of multiple primary antibodies of the same host species or isotype without the problem/concern of crosstalk, thereby greatly enabling multiplexing potential ^8^. One of the best-known mIHC TSA-based methods is Opal™ IHC kit, which is part of PerkinElmer’s Phenoptics™ Research Solution for Cancer Immunology and Immunotherapy. Opal™ uses tyramide-conjugated probes to identify several antigens on tissues and currently can detect up to seven markers simultaneously.

In recent years, a plethora of image analysis software has been developed. Led by ImageJ, there is now a selection of tools such as ImageJ-Fiji, Icy, and CellProfiler to perform image analysis in multiple disciplines ^9– 11^. However, none of these applications can be used for the visualisation and analysis of whole slide images (WSI) and large 2D data. For this reason, there is an increasing interest in developing software designed for digital pathology uses. Visiopharm, Definiens Inc., Halo (Indica Labs), Quantcenter (3DHistech), or open source Qupath ^12^ can be used to analyse WSI data. Overall, these systems share features that provide accurate tissue segmentation and spectral deconvolution for fluorophores and chromogens. Algorithms are used to train the software to classify cells. For example, hue value/width, intensity threshold scoring, morphology/geometrical characterisation, pixel-count threshold, and colour saturation are some of the features that can be used to characterise cells in different tissue areas. Even though artificial intelligence (AI) is already used in radiology and cardiology for image-based diagnosis ^13^, its application to digital pathology is challenging. Currently, the application of AI in digital pathology is confined to several research studies and dedicated companies. Among the areas where AI is already being used are education (teaching at conferences and training of pathologists), quality assurance (teleconsulting and gauging inter- and intra-observer and proficiency testing), clinical diagnosis, and image analysis ^14– 17^.

IHC combined with digital pathology software has the potential to benefit both basic and clinical research (see applications below).

## Applications of digital pathology in translational cancer research

### Assessment of histological and morphological features of tumours

To date, analysis of histological features by haematoxylin and eosin (H&E) is the primary approach and the gold-standard for pathologists for diagnostic purposes in the clinical setting. The use of digital pathology aids clinicians in the identification of new histological areas. Furthermore, digital pathology contributes to standardising phenotypic features linked to malignant phenotypes ^18– 21^.

For instance, skin cutaneous melanoma (SKCM) is one of the most heterogeneous cancers. Pathologists can describe multiple morphological features in the same section ^22^. Previous studies integrated genetic and morphological features ^18^. Interestingly, our group has reported an enrichment in melanoma-rounded cells that harbour high myosin II activity (as measured by p-MLC2) in the invasive fronts of mouse and human tumours ^23– 27^. Indeed, MLC2 is a substrate of Rho-associated kinase (ROCK), one of the major drivers of cancer invasion and a key regulator of actin organisation ^20, 28^. We have associated this morphological feature and high p-MLC2 levels to a very invasive amoeboid-rounded phenotype *in vitro* ^23– 27, 29^ and in combination with intravital imaging studies ^24, 25^.

In all of our previous studies, cancer cell morphology was visually assessed at 20× in the bulk of the tumour using different fields of view (FOVs) ^25– 27^. The visual assessment was based on a scoring method where cancer cells can range from 0 to 3: 0, round morphology; 1, ovoid; 2, elongated; and 3, spindle ^18^. These values then could range from 0 (rounded) to 300 (fully spindle tumour cell) including all intermediate morphologies in between.

The use of digital pathology could overcome inter-observer variability, allows for a more robust analysis and adding new measurements to the analysis. On the other hand, conventional 2D histology provides planar information that, until now, has proved to be valuable diagnostic tools. Of note, 2D assessment of phenotypic features in cancer, such as morphology, angiogenesis, or perineural invasion, are inevitably associated with some level of analytical error. Therefore, obtaining 3D tissue architecture information is still challenging for pathologists but may lead to numerous applications and to the precise description of the complex TME ^30^. For instance, Ourselin *et al.* reported a 3D reconstruction method from consecutive H&E sections to rebuild the microvasculature and correct previous problems ^31^.

### Scoring methods in IHC

As mentioned before, IHC has been used for many decades, but quantification of IHC samples has poor reproducibility ^32^. Histoscore (H-score) is one of the most accepted scoring systems ^33^. H-score is a semi-quantitative assessment of both the intensity of staining (graded as 0, non-staining; 1, weak; 2, median; or 3, high) and the percentage of positive cells. Other methodologies to quantify IHC include the Allred score used to quantify the expression of oestrogen-receptors in breast carcinomas ^34^ or the HER-2 score that gives a score from 0 to 3 and measures the amount of HER2 receptor protein ^35^.

However, since WSI and digital methods have become available, more standardised approaches will overcome reproducibility issues inherent to individual observer bias. Such software can set up thresholds for each of the scores (0–3), emulating the H-score method. Furthermore, by combining machine-educated classifiers to recognise different cell types (cancer, stroma, and immune cells) with a scoring system based on thresholds, it is possible to calculate H-scores in the tumour area. To date, there are few studies using these approaches to standardise quantitative biomarkers that have shown better results than manual methods ^36– 38^. Recently, Acs *et al.* compared three of the most commonly used digital pathology software (Qupath, Quantcenter, and Halo) in order to quantify and assess inter-observer variability for Ki67 in breast carcinoma. Overall, the reproducibility was excellent among all digital pathology platforms, and QuPath showed the lowest intra-digital image analysis variability ^38^.

### Quantification and phenotypic characterisation of immune infiltrates

Understanding the interactions between cancer cells and the TME is an important aspect when predicting therapy responses. In order to characterise TME components, an alternative to flow cytometry (see below) is H&E and IHC. For instance, pre-clinical and translational studies have shown that the percentage of tumour-infiltrating lymphocytes (TILs) is critical to cancer patients’ response to immunotherapies ^37, 39– 43^. Colour separation of histological images (IHC or H&E) has commonly been used to quantify different stains. Most of the platforms—Genie Spectrum (Aperio ePathology Solutions), Image-Pro Plus 3.0 (Media Cybernetics), Halo (Indica Labs), ImageJ, and VMscope (VMscope GmbH)—use colour deconvolution algorithms. Such algorithms were developed to acquire colour information with red–green–blue (RGB) cameras and to calculate the contribution of each colour based on stain-specific RGB absorption ^44^. Recently, our lab has used such approaches by utilising Fiji-ImageJ to quantify F4/80 ^+^CD206 ^+^ macrophages in mouse tissue sections ^27^.

### Extracellular matrix

Another key component of the TME is the extracellular matrix (ECM), a non-cellular three-dimensional macromolecular network that provides structural and biochemical support ^45^. The ECM can be very heterogeneous, and its composition varies both between and within individual tumours. Fibrillar collagen is the most abundant protein in the ECM. Among other ECM components are other collagens, proteoglycans (PGs), and glycoproteins ^46, 47^. ECM can be remodelled during ageing, fibrosis, wound healing, and cancer ^48^. For example, tumours are normally stiffer than their surrounding normal tissue. Such stiffness is induced by ECM deposition, remodelling by fibroblasts, and increased contractility of the transformed epithelium ^49, 50^. Indeed, the importance of the matrisome and ECM stiffness has been recently described in high-grade serous ovarian carcinoma (HGSOC) and other tumour types ^51^. Altered matrix composition and organisation help cancer cells to grow and disseminate, and it is thus an important aspect that needs to be further investigated in tumour biology ^52^.

ECM features are critical to determine drug response in cancer ^53^, and IHC is often used to study matrix constituents ^51^. A number of basic histological techniques can be used to describe the qualitative and quantitative presence and arrangement of matrix components. Gomori’s, Masson’s Trichrome, and Picro Sirius Red are three widespread staining techniques used for the study of the ECM. Indeed, Picro Sirius Red provides highly detailed and contrasted signal derived from connective tissue ^54^. Because of its high informative capability, it is recommended for morphometric assessment of fibrotic responses under cross-polarised light ^54, 55^. The potential to further quantify structures and matrix patterns in tissue sections using digital platforms, such as matrix intensity and anisotropy, can help pathologists and clinical cancer researchers to better understand the structure of tumours.

### Multiplex staining in tissue samples

One of the most revolutionary techniques in tissue imaging to date is multiplex staining using IHC methodologies. Currently, there are limited tools to analyse mIHC. One example is the Phenoptics platform, which allows scientists to adapt and tailor the software to their specific purpose/needs (InForm). However, mIHC-based on SIMPLE requires an advanced knowledge of scripting with Image-J and Cellprofiler ^6^. Currently, there are only a few platforms to perform such analysis, including programs such as Visiopharm. Such software can register and align different markers within the same section. Later, every single cell is segmented and the corresponding positive or negative information for each marker is integrated. Overall, the combination of multiple markers with histological and spatial information creates complex data that require high-throughput data analysis methods. The most common outcomes after the integration of all the data are generally TSNE plots, correlation matrix, and neighbourhood analysis that can guide deeper understanding of the data.

### Machine-learning and artificial intelligence

With the development of AI, deep convolutional neural networks (CNNs) ^56^ have proven to be useful for advancing biomedical imaging, as mentioned above. Application of deep-learning methods and AI in translational studies has been used to investigate 1) how immune infiltrates vary across space within patients at the time of diagnosis ^57^, 2) if the stroma-tumour cell ratio can predict ovarian cancer therapy responses ^58^, and 3) how TME forces shape the plasticity of cancer cells ^21^. Developing these approaches for biomedical research purposes is both exciting and challenging.

## Imaging and flow cytometry for tissue analysis

Flow cytometry is a powerful quantitative technique that allows multiplexing in high-throughput systems (HTS), providing fast and automated sample acquisition (i.e. HTS screening assays). Specifically, multi-parameter flow cytometry (up to 18 colours simultaneously) has enabled high-resolution quantification of cell types, analysis of cell surface and intracellular molecules, immunophenotyping analysis, functional characteristics of different cell populations, and many more. Similarly, mass cytometry can measure >40 parameters simultaneously, necessitating high-dimensional cytometry data analysis with algorithms, such as viSNE (visualize relationships in multidimensional data) and SPADE (spanning-tree progression analysis of density-normalized events). This allows complex data analysis and visualisation of different cell features ^59, 60^. Flow cytometry, in general, has found numerous applications not only in basic research but also in fields like diagnostics and clinical pathology, with a fundamental role in immunology. However, the main limitation of such technology is the use of single-cell suspensions. Therefore, any analysis requires enzymatic digestion or mechanical dissociation of the tissue during sample preparation. Moreover, flow cytometry does not allow spatial information of cell populations of interest within the tissue, along with cell number loss due to tissue processing ^61^. Any cancer cell–non cancer cell interactions (i.e. relationship between tumours cells and immune infiltrates) or information regarding tissue architecture is not possible with flow cytometry ^61^. To address these limitations, a new technology that combines imaging and flow cytometry has been developed ^62, 63^.

Indeed, the so-called field of imaging cytometry is rapidly evolving. In contrast to conventional flow cytometry, imaging offers information about cell structure, size, morphology, and location of cells within the tissue. Based on the sample (cell suspension, adherent cells, or tissue specimens), flow-based (represented by the ImageStream) and solid-phase imaging cytometry have been defined as the two categories of imaging cytometry ^64^. For the purpose of this review, we will focus on the latter category only.

## Laser scanning cytometry

The first solid-phase imaging cytometry, known as laser scanning cytometry (LSC), was developed by Louis Kamentsky and became commercially available by CompuCyte Corporation (acquired by Thorlabs Inc. in 2013), pioneering the field of automated quantitative imaging cytometry ^62, 63^.

From a technical point of view, LSC couples high-sensitivity and accurate signal quantification, utilising flow cytometry advantages of laser excitation and photomultiplier tubes (PMTs), with the spatial resolution of microscopy. For a representative list of the most common imaging cytometers, please see Henriksen *et al.* ^64^. Prototypically, the laser scanning beam was derived from an argon ion laser and later also a helium–neon laser and a violet diode laser that were all combined at a dichroic mirror and guided to a second dichroic mirror to reflect the laser wavelengths ^62, 65^. The laser beam is directed to a computer-controlled scan mirror and passes through a scan lens and then to the microscope objective. Scattered light is collected by the condenser lens of the microscope and directed to solid-state photosensors. The appropriate PMTs are used to detect laser-excited epi-illumination fluorescence. The photosensor and PMT signals are digitised to create pixel values and are gathered into images ^65^. As the beam is scanned over the sample in one axis (y), the stage is moved by the computer-controlled motor in the *x*-axis, generating x–y coordinates ^66^. Nowadays, to allow multiplexing, an LSC is equipped with up to four lasers from six wavelengths in total: 405 nm, 488 nm, 532 nm, 594 nm, 561 nm, and 633 nm ^67^. It is worth mentioning that in addition to PMT-based LSC, charge-coupled device (CCD)-based systems also exist. The latter comprises a dense array of sensors employing widefield illumination. By contrast, PMTs are usually combined with laser spot-scanning to provide high dynamic range. They also have a higher bandwidth and lower signal-to-noise ratio compared to CCD-based systems ^64, 68^. Comparison between LSC and other imaging technologies, such as laser confocal microscopy or conventional flow cytometry, is beyond the scope of this review. For detailed reviews, see 65, 68.

Given that LSC is a slide-based technology, it offers many advantages. Cell morphology, precise anatomical information, cell–cell interactions *in situ*, signalling, and functional and phenotypic characterisation simultaneously with visualisation of cells of interest are some of the main applications of LSC ^61, 65^. Other common applications involve cell cycle and DNA damage analysis and kinetic studies ^67^. The areas that have greatly benefited from LSC are immunology, cytogenetics, diagnostics, and clinical pathology. In contrast to inter-examiners’ reproducibility and the technical limitations of conventional IHC that have been mentioned above, LSC offers objective and accurate quantification in an automated and time-lapse manner. For this reason, histological sections, fine-needle aspirates, patient and healthy volunteers’ swabs, and cytospins have all been widely employed for LSC. Specifically, a comparative study of LSC versus IHC analysis of melanoma-associated antigen recognised by T cells (MART-1), glycoprotein 100 (gp100), and HLA-A2 expression in fine-needle aspirates has highlighted the accuracy of LSC ^69^. Importantly, LSC could allow the discrimination of heterogeneous melanoma antigen expression in subsets of melanoma cells within the same lesion ^69^. On the other hand, the so-called “tissue maps” can be created from the x–y coordinates, allowing the quantification and visualisation of molecular profiles ^65^.

Of note, chromatic or fluorescent dyes can be used for tissue staining ^64^. There are four types of contour accurately defining single cells. A “threshold contour” is defined by nuclear DNA staining, whilst an “integration contour” is defined by cell-surface marker staining. “Peripheral contours” could potentially define the region between the nucleus and cell surface, and finally “background contour” is used for non-cell areas to allow the calculation of the background fluorescence ^65^. Routinely, two- or three-colour chromatically or fluorescently stained tissue specimens, but also multiplexing, are available for the simultaneous evaluation of patients’ biopsies, tissue microarrays (TMAs), and tissue sections ^61^. For example, breast or prostate TMAs have been successfully stained for anti-Her2/3’-diaminobenzidine (DAB)/counterstain with hematoxylin and anti-CD10/DAB/counterstain with hematoxylin, respectively ^64, 70^. In contrast to IHC, LSC can distinguish immune cell populations based on the chromatin condensation ^71^. Another important feature of LSC is the spatial resolution of fluorescence within a cell enabling the nuclear versus cytoplasmic analysis of targets (i.e. NF-κB translocation in the nucleus) ^61^. Furthermore, visualisation and quantification of antigen presentation-related processes, lymphocyte migration, and signalling events can all be dissected by LSC ^65, 72^. Overall, analysis of the immune response *in situ* is improved aided by the use of LSC ^65^.

Moreover, given that the slide position on the stage is recorded, relocation and re-staining of the sample are possible, while the laser excitation wavelength can also be changed between different runs ^73^. These features are particularly important, as patient biopsies are precious and can thus be analysed for rare cell populations which exemplifies the versatility of the LSC ^65^.

## Histo-cytometry

On the other hand, it is worth mentioning that a new method for multiplex quantitative image analysis, known as “histo-cytometry”, was developed by Gerner *et al.* in 2012 ^74^. Such an analytical confocal microscopy method allows visualisation and quantification of phenotypically different immune cell populations within murine lymph nodes. Of note, the authors have used this methodology to provide novel information about the distribution of resident and migratory dendritic cell subsets in discrete lymph node microcompartments.

Overall, LSC combines the advantages of multiparameter flow cytometry and quantitative data analysis with the visualisation and morphometrics offered by imaging to provide high sensitivity and dynamic range. Dissection of TME components, proliferation and apoptosis of cells alongside accurate anatomical information for tumour cells, immune infiltrates, blood vessels, is possible using LSC. Future advances in software options for LSC, in addition to the development of more methods combining imaging and flow cytometry, will allow *in vivo* screening in real time.

## Intravital microscopy

Non-linear optical microscopy has seen great progress since the development of two-photon laser-scanning microscopy, the availability of *in vivo* fluorescent labelling for targets of interest, and the advancement of genetic tools to modify gene expression ^75, 76^. Specifically, intravital imaging using multi-photon microscopy has proved to be an invaluable technique to image many biological processes *in vivo* at great depth and high resolution ^77^. In fact, such an imaging tool has provided flexibility in capturing cell–cell interactions, tissue dynamics and architecture, tumour–host interactions, cancer cell migration, and the dynamics of metastatic dissemination ^78, 79^. The principles of intravital imaging are beyond the scope of this review. For detailed reviews on this topic, please see 77, 80, 81.

Great work has been done by several groups in visualising ECM, tumour cells, immune infiltrates, and other TME components in live rodents. Among the research areas that intravital imaging has shown great potential is cancer cell migration an indispensable step during metastasis ^78^. Directed cell migration involves (a) individual cell migration (rounded-amoeboid and elongated-mesenchymal) and (b) collective migration as multicellular groups, such as cell sheets or strands ^82^. *In vivo* intravital imaging of xenografts (nude mice injected in the mammary fat with rat MTLn3 mammary carcinoma cells) has shown that breast cancer cells migrate and invade using the collective mode of motility in order to enter lymphatic vessels ^83^. In contrast, within the same study, individually migrating breast cancer cells using the amoeboid-rounded type of motility were shown to enter the bloodstream for metastatic dissemination ^83^. On the other hand, multicellular streaming (two to three cells co-migrating in a directional fashion) of human breast cancer cells has been reported to be correlated with a vascularised microenvironment and intravasation in two orthotopic xenograft models ^84^. Of note, macrophages were shown to be necessary for multicellular streaming in those breast tumours ^84^. In fact, intravital imaging has revealed that breast cancer cells interact with macrophages in a paracrine manner to co-migrate *in vivo* in a transgenic mouse model of breast cancer ( *MMTV-PyMT*) ^85^. Strikingly, intravital imaging in melanoma xenografts together with histopathological analysis of melanoma patients’ biopsies revealed that the invasive fronts of melanomas are enriched in rounded-amoeboid cancer cells ^23, 24, 26, 27, 86^. On the other hand, physical cues from the surrounding TME are important in determining the invasion mode of cancer cells ^87^. Indeed, dimension (2D or 3D), density, orientation, porosity, and stiffness (rigidity) of the ECM can altogether affect the invasion strategy of cancer cells ^88^. Intravital imaging and histopathological analysis have shown that tissue structures can act as a guide for both single cell and collective cell migration strategies ^88^.

Therefore, it becomes apparent that cancer cell migration, from detachment from the primary tumour to metastatic dissemination, is a process that should be investigated further considering the complex bidirectional interactions of cancer cells with their surrounding microenvironment. In fact, cancer cells have been shown to migrate along ECM fibres using the amoeboid-rounded type of movement ^78^. Metastasis is very complex and includes a variety of processes such as invasion, intravasation, and extravasation, all of which can be visualised thanks to the advances in intravital microscopy. Indeed, studies have described differences in cancer cell motility and the tumour microenvironment in primary and metastatic tumours ^89^. For example, during intravasation, metastatic versus primary mammary adenocarcinoma cells have been shown to move using the amoeboid-rounded type of intact single cell migration towards blood vessels ^89^. Similarly, non-metastatic cells tend to undergo fragmentation when interacting with blood vessels ^85^. On the other hand, ECM fibres assemble on blood vessels in mammary tumours ^78^. All of these important observations regarding the metastatic cascade were shown using intravital imaging approaches.

Nevertheless, inherent motion, such as respiratory and cardiac motion, of live animals represents a major challenge and lowers the resolution and the area being imaged. To circumvent this limitation, Condeelis’ group has combined mosaicked-stitched imaging with intravital imaging of live tissues to generate a new method—large-volume, high-resolution intravital imaging (LVHR-IV)—thereby developing a new approach for tissue stabilisation ^90^. In general, advancement in fluorescent probes, software, and data analysis tools and tissue stabilisation techniques have addressed several challenges in intravital microscopy.

On the other hand, intravital imaging has provided great insight into the mechanisms of therapy response and resistance in cancer ^79^. For example, BRAF-mutant melanoma cells have been reported to develop resistance to BRAF inhibition *in vivo* due to “paradoxical” activation of melanoma-associated fibroblasts in the TME ^91^. Additionally, intravital spinning disk confocal microscopy has visualised the interaction between tumour-specific T cells and dendritic cells intratumorally, improving our understanding in immunotherapy responses ^92^. For a detailed review on intravital imaging applications on tumour–host interactions and therapy response, please see 79.

Overall, our knowledge about the stages of cancer cell metastasis, cancer cell morphology and motility, tumour heterogeneity, and tumour–stroma interactions have all been significantly improved upon using intravital imaging.

Nevertheless, intravital imaging has several limitations compared to *in vivo* multimodality imaging. Specifically, multimodal imaging techniques, such as single-photon emission computed tomography (SPECT)-CT, positron emission tomography–computed tomography (PET-CT), and magnetic resonance imaging (MRI) scans, have proved to be invaluable diagnostic tools. These are routinely used in the clinic and have no penetration depth limit ^93^. In fact, the bio-distribution of therapeutic agents can be better examined using such macroscopic imaging tools compared to intravital imaging. The latter, by contrast, provides anatomical and physiological information at a molecular and subcellular level. The applications of multimodal imaging technologies will not be discussed here. For a detailed review on the above and pre-clinical and clinical imaging in cancer, see 93, 94. One of the main challenges of intravital imaging is associated with the light absorption and scattering and thus potential low in-focus light emission and out-of-focus light from the tissue of interest ^95^. Despite intravital microscopy being powerful for live cell tracking, it is limited by the duration of tracking and area of imaging ^95^. Hence, the entire course of metastasis cannot be monitored by intravital imaging.

## Other cancer tissue imaging modalities

Other techniques in the field that have contributed to the progress of cancer research include light-sheet fluorescence microscopy (LSFM). LSFM is a technique with an intermediate-to-high optical resolution, but good optical sectioning capabilities and high speed. LSFM can efficiently visualise large tissue samples in three dimensions ^96, 97^, and volumetric 3D imaging can uncover detailed information about the inner landscape of tumours, which can improve cancer diagnosis and therapy ^98^. It also shortens the pathological diagnosis to minutes while the patient is still in the operating room. Whole-body imaging in the medical context can also be applied using SPECT or PET in combination with appropriate contrast agents in order to identify cancer cells *in vivo* ^93, 99^. For preclinical studies, cancer cells can be engineered to express reporters, enabling *in vivo* cancer cell tracking and 3D tomography ^100^. However, fewer imaging tracers have been translated to the clinic because of several bottlenecks (specificity, selectivity, cost, etc.). On the other hand, label-free and high-resolution optical microscopes that can directly identify and image native biomolecules are available. Mass spectrometry imaging (MSI) uses ionised molecules to collect a mass spectrum at each pixel of the tissue section. MSI has the capacity to determine the relative intensity and spatial distribution of several hundreds of compounds from cells and tissue while retaining important spatial information ^101^. MSI has been used for the molecular analysis of cancerous cells and tissues with the aim of identifying tumour margins ^102^. MSI has also been used to classify primary tumour tissues with regard to chemo-response and metastatic status ^103^ and to identify diagnostic and prognostic markers. Furthermore, MSI has been successfully employed to study drug response and resistance rates ^104^. On the other hand, Hyperspectral imaging (HSI) is a hybrid modality that combines imaging and spectroscopy. By collecting spectral information at each pixel of a 2D detector array, HSI generates a 3D dataset of spatial and spectral information, known as hypercube ^105^. Therefore, HSI offers great potential for non-invasive disease diagnosis and surgical guidance. Advanced non-linear imaging modalities like coherent anti-Stokes Raman scattering (CARS) and stimulated Raman scattering (SRS) microscopies have been proposed to improve spatial resolution (up to 130 nm). It is therefore a useful imaging approach that enables the generation of images through endogenous chemical species already present in biological tissues ^106^.

## Conclusions and outlook

Great progress has been made towards dissecting spatiotemporal processes in tissues thanks to an array of advances in microscopy and image analysis which are all summarized in Table 1. We have reported multiple applications of H&E, IHC, and imaging flow cytometry in histological sections. Combining these techniques with new digital pathology platforms has great potential to make faster and more robust observations. Using complementary imaging techniques, such as intravital imaging or LSC combined with multiplex IHC and deep learning methods of the same tissues, would provide more detailed dynamic and molecular information regarding how the TME controls tumorigenesis and metastasis.

**Table 1.** Summary of the three main techniques for imaging tissues.

	BASIC PRINCIPLES	PROS	CONS
**MULTIPLEX** **IMMUNOHISTOCHEMISTRY (IHC)**	Protein detection based on antigen–antibody specificity	Spatial and histological information	Limited antigen detection
	Chromogen or fluorescence- based visualisation	Accessible and affordable	Tissue integrity compromised after several antigen retrievals in multiplexing methods
	Can be performed for multiplex staining. Several methods have been reported. The most common is based on the stripping– reprobing principle	Relatively fast method	Possible spectral overlap in fluorescent multiplex IHC
	Analysis of the tissue section can be performed in several platforms: ImageJ, CellProfiler, Qupath, Halo, Visiopharm, Definiens, etc.	Chromogenic multiplex IHC uses same reagents as conventional IHC	May need multispectral microscope (N >5 colours), which increases cost- effectiveness
**LASER SCANNING CYTOMETRY (LSC)**	Uses laser excitation and photomultiplier tubes (PMTs)	Morphological and spatial information compared to traditional flow cytometry	Less accessible than general IHC procedures
	Samples are retained and analysed on a solid support, such as a slide	High sensitivity and accuracy due to PMTs	Time-consuming and difficult application to clinical practice
	The LSC slide and laser beam are moved under computer control	Objective and accurate quantification in an automated and time- dependent manner	Spectral overlap of fluorochromes when performing multiplexing
**INTRAVITAL MICROSCOPY**	Two-photon excitation microscopy, in which the excitation wavelength is shorter than emission wavelengths	Infrared minimises scattering within tissue	Visualisation limited by fluorescent labels available
	Uses near-infrared excitation light to excite fluorescent dyes	Multiphoton absorption suppresses the background signal	Variability in homogeneity and transparency of different tissues can affect the imaging
	Availability to image *in vivo* tissue	Tissue penetration and reduced photobleaching	Limited access, challenging technology, and limited to preclinical studies

## References

[1] Nakane PK Pierce GB Jr: Enzyme-labeled antibodies for the light and electron microscopic localization of tissue antigens. *J Cell Biol.* 1967;33(2):307–18. 10.1083/jcb.33.2.307 6046571 PMC2108346

[2] Coons AH Creech HJ Jones RN: Immunological Properties of an Antibody Containing a Fluorescent Group. *Exp Biol Med.* 1941;47(2):200–2. 10.3181/00379727-47-13084P

[3] Pantanowitz L Valenstein PN Evans AJ: Review of the current state of whole slide imaging in pathology. *J Pathol Inform.* 2011;2:36. 10.4103/2153-3539.83746 21886892 PMC3162745

[4] Teruya-Feldstein J: The immunohistochemistry laboratory: looking at molecules and preparing for tomorrow. *Arch Pathol Lab Med.* 2010;134(11):1659–65. 21043819 10.5858/2009-0582-RAR1.1

[5] Taylor CR Levenson RM: Quantification of immunohistochemistry--issues concerning methods, utility and semiquantitative assessment II. *Histopathology.* 2006;49(4):411–24. 10.1111/j.1365-2559.2006.02513.x 16978205

[6] Tsujikawa T Kumar S Borkar RN: Quantitative Multiplex Immunohistochemistry Reveals Myeloid-Inflamed Tumor-Immune Complexity Associated with Poor Prognosis. *Cell Rep.* 2017;19(1):203–17. 10.1016/j.celrep.2017.03.037 28380359 PMC5564306

[7] Glass G Papin JA Mandell JW: SIMPLE: a sequential immunoperoxidase labeling and erasing method. *J Histochem Cytochem.* 2009;57(10):899–905. 10.1369/jhc.2009.953612 19365090 PMC2746723

[8] Stack EC Wang C Roman KA: Multiplexed immunohistochemistry, imaging, and quantitation: a review, with an assessment of Tyramide signal amplification, multispectral imaging and multiplex analysis. *Methods.* 2014;70(1):46–58. 10.1016/j.ymeth.2014.08.016 25242720

[9] Sero JE Bakal C: Multiparametric Analysis of Cell Shape Demonstrates that β-PIX Directly Couples YAP Activation to Extracellular Matrix Adhesion. *Cell Syst.* 2017;4(1):84–96.e6. 10.1016/j.cels.2016.11.015 28065575 PMC5289939

[10] Graml V Studera X Lawson JLD: A genomic Multiprocess survey of machineries that control and link cell shape, microtubule organization, and cell-cycle progression. *Dev Cell.* 2014;31(2):227–39. 10.1016/j.devcel.2014.09.005 25373780 PMC4648281

[11] Sailem HZ Sero JE Bakal C: Visualizing cellular imaging data using PhenoPlot. *Nat Commun.* 2015;6:5825. 10.1038/ncomms6825 25569359 PMC4354266

[12] Bankhead P Loughrey MB Fernández JA: QuPath: Open source software for digital pathology image analysis. *Sci Rep.* 2017;7(1):16878. 10.1038/s41598-017-17204-5 29203879 PMC5715110

[13] Jha S Topol EJ: Adapting to Artificial Intelligence: Radiologists and Pathologists as Information Specialists. *JAMA.* 2016;316(22):2353–2354. 10.1001/jama.2016.17438 27898975

[14] Niazi MKK Parwani AV Gurcan MN: Digital pathology and artificial intelligence. *Lancet Oncol.* 2019;20(5):e253–e261. 10.1016/S1470-2045(19)30154-8 31044723 PMC8711251

[15] Esteva A Kuprel B Novoa RA: Dermatologist-level classification of skin cancer with deep neural networks. *Nature.* 2017;542(7639):115–8. 10.1038/nature21056 28117445 PMC8382232

[16] Poplin R Varadarajan AV Blumer K: Prediction of cardiovascular risk factors from retinal fundus photographs via deep learning. *Nat Biomed Eng.* 2018;2(3):158–64. 10.1038/s41551-018-0195-0 31015713

[17] Ehteshami Bejnordi B Veta M Johannes van Diest P: Diagnostic Assessment of Deep Learning Algorithms for Detection of Lymph Node Metastases in Women With Breast Cancer. *JAMA.* 2017;318(22):2199–210. 10.1001/jama.2017.14585 29234806 PMC5820737

[18] Viros A Fridlyand J Bauer J: Improving melanoma classification by integrating genetic and morphologic features. *PLoS Med.* 2008;5(6):e120. 10.1371/journal.pmed.0050120 18532874 PMC2408611

[19] Macenko M Niethammer M Marron JS: A method for normalizing histology slides for quantitative analysis. In *2009 IEEE International Symposium on Biomedical Imaging: From Nano to Macro*,2009;1107–1110. 10.1109/ISBI.2009.5193250

[20] Veta M Pluim JP van Diest PJ: Breast cancer histopathology image analysis: a review. *IEEE Trans Biomed Eng.* 2014;61(5):1400–11. 10.1109/TBME.2014.2303852 24759275

[21] Heindl A Khan AM Rodrigues DN: Microenvironmental niche divergence shapes BRCA1-dysregulated ovarian cancer morphological plasticity. *Nat Commun.* 2018;9(1):3917. 10.1038/s41467-018-06130-3 30254278 PMC6156340

[22] Grzywa TM Paskal W Włodarski PK: Intratumor and Intertumor Heterogeneity in Melanoma. *Transl Oncol.* 2017;10(6):956–75. 10.1016/j.tranon.2017.09.007 29078205 PMC5671412

[23] Orgaz JL Pandya P Dalmeida R: Diverse matrix metalloproteinase functions regulate cancer amoeboid migration. *Nat Commun.* 2014;5:4255. 10.1038/ncomms5255 24963846 PMC4118761

[24] Herraiz C Calvo F Pandya P: Reactivation of p53 by a Cytoskeletal Sensor to Control the Balance Between DNA Damage and Tumor Dissemination. *J Natl Cancer Inst.* 2016;108(1): pii: djv289. 10.1093/jnci/djv289 26464464 PMC4712681

[25] Sanz-Moreno V Gaggioli C Yeo M: ROCK and JAK1 signaling cooperate to control actomyosin contractility in tumor cells and stroma. *Cancer Cell.* 2011;20(2):229–45. 10.1016/j.ccr.2011.06.018 21840487

[26] Cantelli G Orgaz JL Rodriguez-Hernandez I: TGF-β-Induced Transcription Sustains Amoeboid Melanoma Migration and Dissemination. *Curr Biol.* 2015;25(22):2899–914. 10.1016/j.cub.2015.09.054 26526369 PMC4651903

[27] Georgouli M Herraiz C Crosas-Molist E: Regional Activation of Myosin II in Cancer Cells Drives Tumor Progression via a Secretory Cross-Talk with the Immune Microenvironment. *Cell.* 2019;176(4):757–774.e23. 10.1016/j.cell.2018.12.038 30712866 PMC6370915

[28] Riento K Ridley AJ: Rocks: multifunctional kinases in cell behaviour. *Nat Rev Mol Cell Biol.* 2003;4(6):446–56. 10.1038/nrm1128 12778124

[29] Crosas-Molist E Bertran E Rodriguez-Hernandez I: The NADPH oxidase NOX4 represses epithelial to amoeboid transition and efficient tumour dissemination. *Oncogene.* 2017;36(21):3002–14. 10.1038/onc.2016.454 27941881 PMC5354266

[30] Xu Y Pickering JG Nong Z: A Method for 3D Histopathology Reconstruction Supporting Mouse Microvasculature Analysis. *PLoS One.* 2015;10(5):e0126817. 10.1371/journal.pone.0126817 26024221 PMC4449209

[31] Ourselin S Roche A Subsol G: Reconstructing a 3D structure from serial histological sections. *Image Vis Comput.* 2001;19(1–2):25–31. 10.1016/S0262-8856(00)00052-4

[32] Lawrie CH Ballabio E Soilleux E: Inter- and intra-observational variability in immunohistochemistry: a multicentre analysis of diffuse large B-cell lymphoma staining. *Histopathology.* 2012;61(1):18–25. 10.1111/j.1365-2559.2012.04179.x 22372580

[33] Hirsch FR Varella-Garcia M Bunn PA Jr: Epidermal growth factor receptor in non-small-cell lung carcinomas: correlation between gene copy number and protein expression and impact on prognosis. *J Clin Oncol.* 2003;21(20):3798–807. 10.1200/JCO.2003.11.069 12953099

[34] Qureshi A Pervez S: Allred scoring for ER reporting and it's impact in clearly distinguishing ER negative from ER positive breast cancers. *J Pak Med Assoc.* 2010;60(5):350–3. 20527604

[35] Wolff AC Hammond ME Schwartz JN: American Society of Clinical Oncology/College of American Pathologists guideline recommendations for human epidermal growth factor receptor 2 testing in breast cancer. *J Clin Oncol.* 2007;25(1):118–45. 10.1200/JCO.2006.09.2775 17159189

[36] Johansson AC Visse E Widegren B: Computerized image analysis as a tool to quantify infiltrating leukocytes: a comparison between high- and low-magnification images. *J Histochem Cytochem.* 2001;49(9):1073–9. 10.1177/002215540104900902 11511677

[37] Angell HK Gray N Womack C: Digital pattern recognition-based image analysis quantifies immune infiltrates in distinct tissue regions of colorectal cancer and identifies a metastatic phenotype. *Br J Cancer.* 2013;109(6):1618–24. 10.1038/bjc.2013.487 23963148 PMC3776996

[38] Acs B Pelekanou V Bai Y: Ki67 reproducibility using digital image analysis: an inter-platform and inter-operator study. *Lab Invest.* 2019;99(1):107–17. 10.1038/s41374-018-0123-7 30181553

[39] Ropponen KM Eskelinen MJ Lipponen PK: Prognostic value of tumour-infiltrating lymphocytes (TILs) in colorectal cancer. *J Pathol.* 1997;182(3):318–24. 10.1002/(SICI)1096-9896(199707)182:3<318::AID-PATH862>3.0.CO;2-6 9349235

[40] Hanahan D Coussens LM: Accessories to the crime: functions of cells recruited to the tumor microenvironment. *Cancer Cell.* 2012;21(3):309–22. 10.1016/j.ccr.2012.02.022 22439926

[41] Neubert NJ Schmittnaegel M Bordry N: T cell-induced CSF1 promotes melanoma resistance to PD1 blockade. *Sci Transl Med.* 2018;10(436):pii: eaan3311. 10.1126/scitranslmed.aan3311 29643229 PMC5957531

[42] Pai CS Huang JT Lu X: Clonal Deletion of Tumor-Specific T Cells by Interferon-γ Confers Therapeutic Resistance to Combination Immune Checkpoint Blockade. *Immunity.* 2019;50(2):477–492.e8. 10.1016/j.immuni.2019.01.006 30737146 PMC6886475

[43] Sosman JA Kim KB Schuchter L: Survival in BRAF V600-mutant advanced melanoma treated with vemurafenib. *N Engl J Med.* 2012;366(8):707–14. 10.1056/NEJMoa1112302 22356324 PMC3724515

[44] Ruifrok AC Johnston DA: Quantification of histochemical staining by color deconvolution. *Anal Quant Cytol Histol.* 2001;23(4):291–9. 11531144

[45] Bonnans C Chou J Werb Z: Remodelling the extracellular matrix in development and disease. *Nat Rev Mol Cell Biol.* 2014;15(12):786–801. 10.1038/nrm3904 25415508 PMC4316204

[46] Hynes RO Naba A: Overview of the matrisome--an inventory of extracellular matrix constituents and functions. *Cold Spring Harb Perspect Biol.* 2012;4(1):a004903. 10.1101/cshperspect.a004903 21937732 PMC3249625

[47] Wolf K Alexander S Schacht V: Collagen-based cell migration models *in vitro* and *in vivo*. *Semin Cell Dev Biol.* 2009;20(8):931–41. 10.1016/j.semcdb.2009.08.005 19682592 PMC4021709

[48] Frantz C Stewart KM Weaver VM: The extracellular matrix at a glance. *J Cell Sci.* 2010;123(Pt 24):4195–200. 10.1242/jcs.023820 21123617 PMC2995612

[49] Butcher DT Alliston T Weaver VM: A tense situation: forcing tumour progression. *Nat Rev Cancer.* 2009;9(2):108–22. 10.1038/nrc2544 19165226 PMC2649117

[50] Grasset EM Bertero T Bozec A: Matrix Stiffening and EGFR Cooperate to Promote the Collective Invasion of Cancer Cells. *Cancer Res.* 2018;78(18):5229–42. 10.1158/0008-5472.CAN-18-0601 30026329

[51] Pearce OMT Delaine-Smith RM Maniati E: Deconstruction of a Metastatic Tumor Microenvironment Reveals a Common Matrix Response in Human Cancers. *Cancer Discov.* 2018;8(3):304–19. 10.1158/2159-8290.CD-17-0284 29196464 PMC5837004

[52] Pickup MW Mouw JK Weaver VM: The extracellular matrix modulates the hallmarks of cancer. *EMBO Rep.* 2014;15(12):1243–53. 10.15252/embr.201439246 25381661 PMC4264927

[53] Vennin C Chin VT Warren SC: Transient tissue priming via ROCK inhibition uncouples pancreatic cancer progression, sensitivity to chemotherapy, and metastasis. *Sci Transl Med.* 2017;9(384): pii:eaai8504. 10.1126/scitranslmed.aai8504 28381539 PMC5777504

[54] James J Bosch KS Aronson DC: Sirius red histophotometry and spectrophotometry of sections in the assessment of the collagen content of liver tissue and its application in growing rat liver. *Liver.* 1990;10(1):1–5. 10.1111/j.1600-0676.1990.tb00428.x 2308475

[55] Junqueira LC Bignolas G Brentani RR: Picrosirius staining plus polarization microscopy, a specific method for collagen detection in tissue sections. *Histochem J.* 1979;11(4):447–55. 10.1007/bf01002772 91593

[56] LeCun Y Bengio Y Hinton G: Deep learning. *Nature.* 2015;521(7553):436–44. 10.1038/nature14539 26017442

[57] Zhang AW McPherson A Milne K: Interfaces of Malignant and Immunologic Clonal Dynamics in Ovarian Cancer. *Cell.* 2018;173(7):1755–1769.e22. 10.1016/j.cell.2018.03.073 29754820

[58] Lan C Li J Huang X: Stromal cell ratio based on automated image analysis as a predictor for platinum-resistant recurrent ovarian cancer. *BMC Cancer.* 2019;19(1):159. 10.1186/s12885-019-5343-8 30777045 PMC6380057

[59] Saeys Y van Gassen S Lambrecht BN: Computational flow cytometry: Helping to make sense of high-dimensional immunology data. *Nat Rev Immunol.* 2016;16(7):449–62. 10.1038/nri.2016.56 27320317

[60] Spitzer MH Nolan GP: Mass Cytometry: Single Cells, Many Features. *Cell.* 2016;165(4):780–91. 10.1016/j.cell.2016.04.019 27153492 PMC4860251

[61] Darzynkiewicz Z Bedner E Li X: Laser-scanning cytometry: A new instrumentation with many applications. *Exp Cell Res.* 1999;249(1):1–12. 10.1006/excr.1999.4477 10328948

[62] Kamentsky LA Kamentsky LD Fletcher JA: Methods for automatic multiparameter analysis of fluorescence in situ hybridized specimens with a laser scanning cytometer. *Cytometry.* 1997;27(2):117–25. 10.1002/(sici)1097-0320(19970201)27:2<117::aid-cyto3>3.0.co;2-d 9012378

[63] Kamentsky LA Kamentsky LD: Microscope-based multiparameter laser scanning cytometer yielding data comparable to flow cytometry data. *Cytometry.* 1991;12(5):381–7. 10.1002/cyto.990120502 1935453

[64] Henriksen M Miller B Newmark J: Laser scanning cytometry and its applications: A pioneering technology in the field of quantitative imaging cytometry. *Methods Cell Biol.* 2011;102:161–205. 10.1016/B978-0-12-374912-3.00007-9 21704839

[65] Harnett MM: Laser scanning cytometry: Understanding the immune system *in situ*. *Nat Rev Immunol.* 2007;7(11):897–904. 10.1038/nri2188 17917673

[66] Reeve L Rew DA: New technology in the analytical cell sciences: The laser scanning cytometer. *Eur J Surg Oncol.* 1997;23(5):445–50. 10.1016/s0748-7983(97)93728-8 9393576

[67] Henriksen M: Quantitative imaging cytometry: Instrumentation of choice for automated cellular and tissue analysis. *Nat Meth.* 2010;7:i–ii. 10.1038/nmeth.f.302

[68] Han Y Gu Y Zhang AC: Review: Imaging technologies for flow cytometry. *Lab Chip.* 2016;16(24):4639–47. 10.1039/c6lc01063f 27830849 PMC5311077

[69] Mocellin S Fetsch P Abati A: Laser Scanning Cytometry Evaluation of MART-1, gp100, and HLA-A2 Expression in Melanoma Metastases. *J Immunother.* 2001;24(6):447–58. 10.1097/00002371-200111000-00002 11759068

[70] Pozarowski P Holden E Darzynkiewicz Z: Laser scanning cytometry: Principles and applications. *Methods Mol Biol.* 2006;319:165–92. 10.1007/978-1-59259-993-6_8 16719355 PMC3892962

[71] Bedner E Burfeind P Gorczyca W: Laser scanning cytometry distinguishes lymphocytes, monocytes, and granulocytes by differences in their chromatin structure. *Cytometry.* 1997;29(3):191–6. 10.1002/(SICI)1097-0320(19971101)29:3<191::AID-CYTO1>3.0.CO;2-F 9389435

[72] Garside P Ingulli E Merica RR: Visualization of specific B and T lymphocyte interactions in the lymph node. *Science.* 1998;281(5373):96–9. 10.1126/science.281.5373.96 9651253

[73] Kamentsky LA Burger DE Gershman RJ: Slide-based laser scanning cytometry. *Acta Cytol.* 1997;41(1):123–43. 10.1159/000332315 9022736

[74] Gerner MY Kastenmuller W Ifrim I: Histo-cytometry: A method for highly multiplex quantitative tissue imaging analysis applied to dendritic cell subset microanatomy in lymph nodes. *Immunity.* 2012;37(2):364–76. 10.1016/j.immuni.2012.07.011 22863836 PMC3514885

[75] Denk W Strickler JH Webb WW: Two-photon laser scanning fluorescence microscopy. *Science.* 1990;248(4951):73–6. 10.1126/science.2321027 2321027

[76] Helmchen F Denk W: Deep tissue two-photon microscopy. *Nat Meth.* 2005;2(12):932–40. 10.1038/nmeth818 16299478

[77] Weigert R Sramkova M Parente L: Intravital microscopy: A novel tool to study cell biology in living animals. *Histochem Cell Biol.* 2010;133(5):481–91. 10.1007/s00418-010-0692-z 20372919 PMC3027219

[78] Condeelis J Segall JE: Intravital imaging of cell movement in tumours. *Nat Rev Cancer.* 2003;3(12):921–30. 10.1038/nrc1231 14737122

[79] Alexander S Weigelin B Winkler F: Preclinical intravital microscopy of the tumour-stroma interface: Invasion, metastasis, and therapy response. *Curr Opin Cell Biol.* 2013;25(5):659–71. 10.1016/j.ceb.2013.07.001 23896198

[80] Masedunskas A Milberg O Porat-Shliom N: Intravital microscopy: A practical guide on imaging intracellular structures in live animals. *Bioarchitecture.* 2012;2(5):143–57. 10.4161/bioa.21758 22992750 PMC3696059

[81] Williams RM Zipfel WR Webb WW: Multiphoton microscopy in biological research. *Curr Opin Chem Biol.* 2001;5(5):603–8. 10.1016/S1367-5931(00)00241-6 11578936

[82] Friedl P Wolf K: Plasticity of cell migration: A multiscale tuning model. *J Cell Biol.* 2010;188(1):11–9. 10.1083/jcb.200909003 19951899 PMC2812848

[83] Giampieri S Manning C Hooper S: Localized and reversible TGFbeta signalling switches breast cancer cells from cohesive to single cell motility. *Nat Cell Biol.* 2009;11(11):1287–96. 10.1038/ncb1973 19838175 PMC2773241

[84] Patsialou A Bravo-Cordero JJ Wang Y: Intravital multiphoton imaging reveals multicellular streaming as a crucial component of *in vivo* cell migration in human breast tumors. *Intravital.* 2013;2(2):e25294. 10.4161/intv.25294 25013744 PMC3908591

[85] Wyckoff JB Jones JG Condeelis JS: A critical step in metastasis: *in vivo* analysis of intravasation at the primary tumor. *Cancer Res.* 2000;60(9):2504–11. 10811132

[86] Sanz-Moreno V Gadea G Ahn J: Rac activation and inactivation control plasticity of tumor cell movement. *Cell.* 2008;135(3):510–23. 10.1016/j.cell.2008.09.043 18984162

[87] Pandya P Orgaz JL Sanz-Moreno V: Modes of invasion during tumour dissemination. *Mol Oncol.* 2017;11(1):5–27. 10.1002/1878-0261.12019 28085224 PMC5423224

[88] Friedl P Alexander S: Cancer invasion and the microenvironment: Plasticity and reciprocity. *Cell.* 2011;147(5):992–1009. 10.1016/j.cell.2011.11.016 22118458

[89] Wang W Wyckoff JB Frohlich VC: Single cell behavior in metastatic primary mammary tumors correlated with gene expression patterns revealed by molecular profiling. *Cancer Res.* 2002;62(21):6278–88. 12414658

[90] Entenberg D Pastoriza JM Oktay MH: Time-lapsed, large-volume, high-resolution intravital imaging for tissue-wide analysis of single cell dynamics. *Methods.* 2017;128:65–77. 10.1016/j.ymeth.2017.07.019 28911733 PMC5659295

[91] Hirata E Girotti MR Viros A: Intravital imaging reveals how BRAF inhibition generates drug-tolerant microenvironments with high integrin β1/FAK signaling. *Cancer Cell.* 2015;27(4):574–88. 10.1016/j.ccell.2015.03.008 25873177 PMC4402404

[92] Engelhardt JJ Boldajipour B Beemiller P: Marginating dendritic cells of the tumor microenvironment cross-present tumor antigens and stably engage tumor-specific T cells. *Cancer Cell.* 2012;21(3):402–17. 10.1016/j.ccr.2012.01.008 22439936 PMC3311997

[93] Weissleder R Pittet MJ: Imaging in the era of molecular oncology. *Nature.* 2008;452(7187):580–9. 10.1038/nature06917 18385732 PMC2708079

[94] Fruhwirth GO Diocou S Blower PJ: A whole-body dual-modality radionuclide optical strategy for preclinical imaging of metastasis and heterogeneous treatment response in different microenvironments. *J Nucl Med.* 2014;55(4):686–94. 10.2967/jnumed.113.127480 24604910 PMC6205625

[95] Pittet MJ Weissleder R: Intravital imaging. *Cell.* 2011;147(5):983–91. 10.1016/j.cell.2011.11.004 22118457 PMC3824153

[96] Huisken J Swoger J Del Bene F: Optical sectioning deep inside live embryos by selective plane illumination microscopy. *Science.* 2004;305(5686):1007–9. 10.1126/science.1100035 15310904

[97] Tainaka K Kubota SI Suyama TQ: Whole-body imaging with single-cell resolution by tissue decolorization. *Cell.* 2014;159(4):911–24. 10.1016/j.cell.2014.10.034 25417165

[98] Nojima S Susaki EA Yoshida K: CUBIC pathology: three-dimensional imaging for pathological diagnosis. *Sci Rep.* 2017;7(1):9269. 10.1038/s41598-017-09117-0 28839164 PMC5571108

[99] Weissleder R Schwaiger MC Gambhir SS: Imaging approaches to optimize molecular therapies. *Sci Transl Med.* 2016;8(355):355ps16. 10.1126/scitranslmed.aaf3936 27605550

[100] Volpe A Kurtys E Fruhwirth GO: Cousins at work: How combining medical with optical imaging enhances *in vivo* cell tracking. *Int J Biochem Cell Biol.* 2018;102:40–50. 10.1016/j.biocel.2018.06.008 29960079 PMC6593261

[101] Schwamborn K Caprioli RM: Molecular imaging by mass spectrometry--looking beyond classical histology. *Nat Rev Cancer.* 2010;10(9):639–46. 10.1038/nrc2917 20720571

[102] Chaurand P Sanders ME Jensen RA: Proteomics in diagnostic pathology: profiling and imaging proteins directly in tissue sections. *Am J Pathol.* 2004;165(4):1057–68. 10.1016/S0002-9440(10)63367-6 15466373 PMC3118833

[103] Bauer JA Chakravarthy AB Rosenbluth JM: Identification of markers of taxane sensitivity using proteomic and genomic analyses of breast tumors from patients receiving neoadjuvant paclitaxel and radiation. *Clin Cancer Res.* 2010;16(2):681–90. 10.1158/1078-0432.CCR-09-1091 20068102 PMC2892225

[104] Arentz G Mittal P Zhang C: Applications of Mass Spectrometry Imaging to Cancer. *Adv Cancer Res.* 2017;134:27–66. 10.1016/bs.acr.2016.11.002 28110654

[105] Lu G Fei B: Medical hyperspectral imaging: a review. *J Biomed Opt.* 2014;19(1):10901. 10.1117/1.JBO.19.1.010901 24441941 PMC3895860

[106] Bi Y Yang C Chen Y: Near-resonance enhanced label-free stimulated Raman scattering microscopy with spatial resolution near 130 nm. *Light Sci Appl.* 2018;7:81. 10.1038/s41377-018-0082-1 30374403 PMC6199294

